# Realization of Circular Economy of 3D Printed Plastics: A Review

**DOI:** 10.3390/polym13050744

**Published:** 2021-02-27

**Authors:** Caihan Zhu, Tianya Li, Mohamedazeem M. Mohideen, Ping Hu, Ramesh Gupta, Seeram Ramakrishna, Yong Liu

**Affiliations:** 1 College of Materials Science and Engineering, Beijing University of Chemical Technology, Beijing 100029, China; 2020200292@mail.buct.edu.cn (C.Z.); 2018400127@mail.buct.edu.cn (T.L.); mohamedazeem1507@gmail.com (M.M.M.); 2 Department of Chemical Engineering, Tsinghua University, Beijing 100084, China; hspinghu@mail.tsinghua.edu.cn; 3 School of Agricultural Sciences and Rural Development, Nagaland University, Medziphema 797106, India; rameshgupta1954@gmail.com; 4 Nanoscience and Nanotechnology Initiative, National University of Singapore, Singapore 11576, Singapore

**Keywords:** 3D printing, biodegradation, catalytic degradation, circular economy

## Abstract

3D printing technology is a versatile technology. The waste of 3D printed plastic products is a matter of concern because of its impact on the circular economy. In this paper, we discuss the current status and problems of 3D printing, different methods of 3D printing, and applications of 3D printing. This paper focuses on the recycling and degradation of different 3D printing materials. The degradation, although it can be done without pollution, has restrictions on the type of material and time. Degradation using ionic liquids can yield pure monomers but is only applicable to esters. The reprocessing recycling methods can re-utilize the excellent properties of 3D printed materials many times but are limited by the number of repetitions of 3D printed materials. Although each has its drawbacks, the great potential of the recycling of 3D printed waste plastics is successfully demonstrated with examples. Various recycling approaches provide the additional possibility of utilizing 3D printing waste to achieve more efficient circular application.

## 1. Introduction

Plastics are the most ubiquitous and versatile material that plays a paramount role in our modern economy. Presently the yearly plastic production is nearly 360 million tons [1], and its use in the past fifty years has increased twenty-fold. It is expected to double in the upcoming two decades. Despite having many socio-economic advantages, plastic’s low reuse rate and immature reprocessing technologies are currently the main problems. For instance, Australia produces three million tons of plastic every year; 95% was discarded as plastic waste after a single use. By 2050, it is presumed that the accumulation of plastic waste will reach 3.4 billion tons, and most of the waste is landfilled, burned, and leaked into the atmosphere and it pollutes the sea, soil, and will worsen nature and its resources [2]. To mitigate climate change and protect our environment, we need a revolutionary transition from today’s take-make-waste system to the concept of a ‘circular economy’ globally.

Take the UK Plastics Pact as an example. In the UK, 44% of plastic packaging is now recycled. Supermarkets and businesses are providing plastic recycling points and enhancing packaging recycling signage to make packaging more recyclable. In 2018, 65% of packaging from convention members was recycled and had an average of 10% recycled content, saving over one million barrels of oil (over 90,000 tons) to produce plastic [3]. In terms of the Directive on Reducing Plastic Products and Plastic Wastes, the UN Environment Program estimates that the cost of damage to the global marine environment is at least $8 billion per year. In the following, in the concept of a circular economy for plastics, the European Commission expects to help avoid 22 billion euros of environmental damage by 2030 and save consumers 6.5 billion euros. The adaptation costs for producers are expected to be only 3.2 billion euros [4].

A circular economy is a kind of economic growth model that takes the efficient use and recycling of resources as the core, ‘reduce, reuse, and resource’ as the principle, low consumption, low emission, and high efficiency as the essential characteristics. It is in line with the concept of sustainable development, which is a fundamental change to the traditional growth model of ‘mass production, mass consumption, and mass waste’. The circular economy is a promising alternative to the traditional linear economy that provides an opportunity to produce pollution-free plastics with profound socio-economic and environmental benefits in all aspects. There are three stages of a circular economy. The first stage is from 1966 to 1992, mainly the germination of the circular economy and the initial exploration stage. The concept of circular economy and the initial thought of circular economy were proposed. From 1992 to 2010 was the second stage of research and formulation of theoretical models of a circular economy. In this stage, the circular economy concept started to become widespread, and people realized the necessity of a circular economy from facts. Since 2010, the circular economy has been implemented in enterprises as the third phase, and it is considered a vital element of the fourth industrial revolution [5]. The circular economy aims to recycle resources based on the 3R principles: reduce, reuse, and recycle. With the 3R principles, ‘recover, redesign, remanufacture’ were added methodologies becoming the 6R principle [6].

The invention of 3D printing has provided additional tools to the printing industry. 3D printing is also referred to as an additive manufacturing technique that allows the construction of complex geometrical structures ranging from micro to macro scale layer by layer through computer aided design to the final 3D object. Compared to the traditional method, 3D printing has tremendous advantages such as fast design and production, cost-effectiveness, ease of access, rapid prototyping, flexible designing, reducing waste, and being environment friendly. The difference from traditional molding processes is shown in Table 1. Depending upon the nature of the input materials, whether solid, liquid, or powder, 3D printing has been classified into various methods such as Stereo Lithography Appearance (SLA) [7,8], Laminated Object Manufacturing (LOM) [9], Fused Deposition Molding (FDM) [10], Selective Laser Sintering (SLS) [11], Three-Dimensional Printing and Gluing (3DP) [12], and their corresponding processing and material requirements were shown in Table 2.

**Figure 1:**
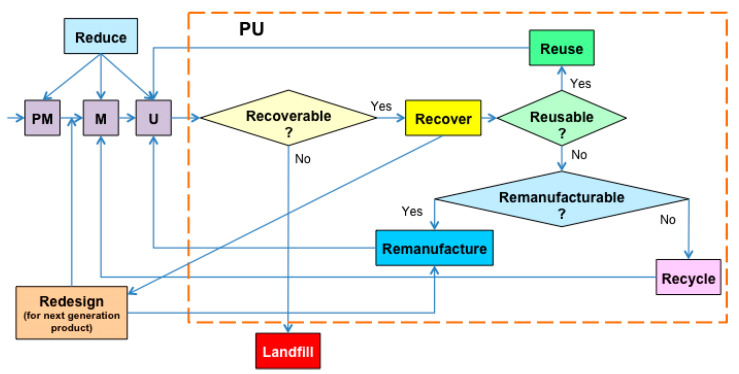
Sequencing of the 6R application within the total life cycle with decision points and multiple closed sloops [6].

Plastics are considered a significant material for 3D printing and must comply with the 6R principle to have sufficient value and utilization. The circular development of 3D printing materials can be implemented mainly from the following aspects, as shown in Figure 1. Both chemical and physical recycling of 3D printed waste plastics were developed so that the waste of 3D printed plastics can be degraded into useful molecules, reused, or rendered harmless.

## 2. Application of 3D Printing

**Figure 2:**
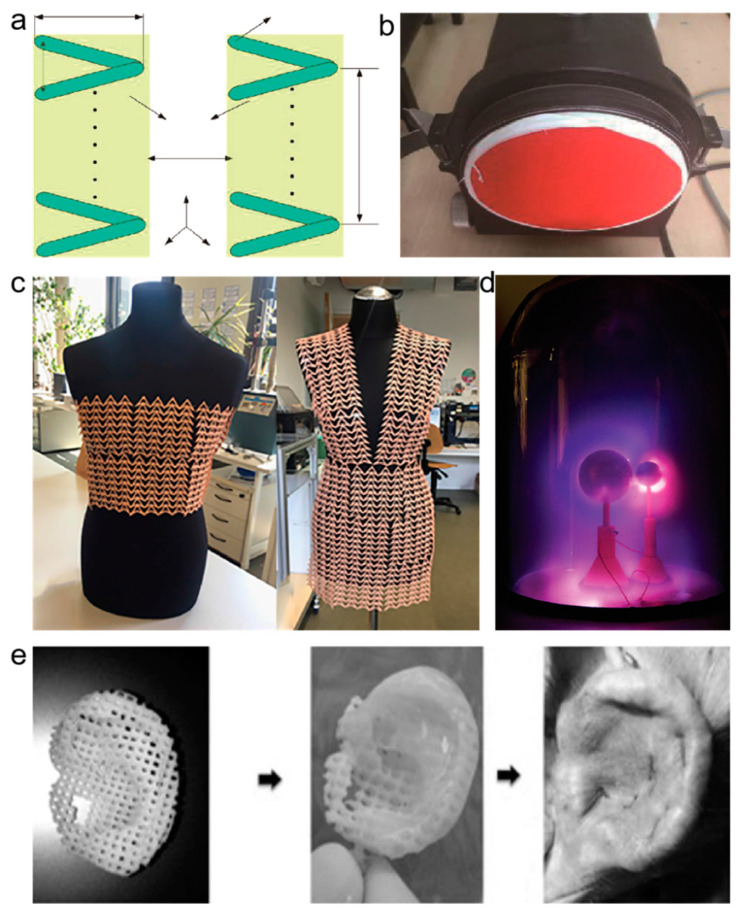
Applications of 3D printing technology in different fields. (**a**) Micro-helix metamaterial structure diagram; (**b**) sound absorption device and the red part is the sound absorption sample produced by 3D printing; (**c**) 3D printed dress; (**d**) 3D printed part used in laboratory; (**e**) the 3D printed part of facial plastic [20,21,22,23].

The application of 3D printing technology was limited to only the production of soft plastics until the mid-2000s. Then its application slowly emerged to be widespread in various fields, as shown in Figure 2. It is one of the most outstanding breakthrough innovations in this century to address the medicine and pharmaceutical research field’s complexity. Since the start of the COVID-19 pandemic, globally, the health care system has faced inadequate demand in medicine and personal protection types of equipment. 3D printed medical devices, including antibacterial bio-cellulose masks produced from biocomposite materials, ventilator valves, and 3D printed test swabs are the pathbreaking innovations to overcome the front line needs in the pandemic scenario. 3D printing has a significant role in biomedicine due to its accurate presentation of complex structures produced from digital models.

3D printing from polymer has gained critical attention in dentistry applications because of its accuracy, precision, and efficiency. Polymer-based biomaterials, like PLA, have been considered the most promising dental materials due to their unique characteristics, including excellent mechanical, physical, and biological properties. For instance, 3D printed models from PLA for education [13]; 3D printed polycaprolactone (PCL) to help the periodontium regeneration [14,15]; 3D printing implants and scaffolds for oral defects repair [16,17].

The toys industry is recently booming with 3D printing technology by manufacturing complex geometrical shapes into attractive 3D toys by utilizing polymers and other kinds of plastic materials—for instance, 3D printing PLA roly-poly toys [18,19]. In terms of clothing, 3D utility has successfully printed PLA into clothing [20] (Figure 2c). Recent research suggests that PLA as a 3D printing material meets the ordinary laboratories’ requirements in strength, precision, vacuum compatibility, and electrical properties [21] (Figure 2d). The precision manufacturing of complex structures can also apply to noise reduction by manufacturing materials into noise reduction structures (Figure 2a,b) [22]. Plastic surgery is ubiquitous globally, and 3D printing can meet customers’ needs [23] (Figure 2e).

To meet the requirements of different fields, 3D printing materials have become the focus of attention. The commercially 3D printed plastic was shown in Table 3. It includes PLA, Acrylonitrile Butadiene Styrene (ABS), PCL, Polyamide (PA), Polycarbonate (PC), etc. The effective reuse of the materials is one of the most important ways to achieve sustainable development, so it is crucial to reuse the printed materials.

## 3. Current Status and Problems of 3D Printing

Studying the countrywide performance in China, 3D printing technology is not yet at a world-class level. Especially for industrial-grade products, the main problems are lack of linkage between precision, stability, materials, etc. There are currently about 130 types of 3D printing materials, mainly classified from polymers, metal, ceramics, and concrete. Among these materials, polymers were mainly utilizing 3D printing material [25]. In 2019, the global 3D printing industry scale reached 11.956 billion dollars, a growth rate of 29.9%, and China’s 3D printing industry scale reached 15.75 billion Yuan [26]. 3D printing has been widespread in biomedical, aviation, defense, toys, jewelry industries, and other end-use sectors in the current scenario.

In contrast to China, several other countries, such as the United States, currently study Ford Freeform Fabrication Technology (F3T) and intend to apply the technology to home appliances, transportation, aviation, defense, etc. India is currently using 3D printing in automobiles, electronics, construction, and the oil industry. Singapore is implementing 3D printing in the medical field, such as prosthetics and porous materials for drug delivery. In Russia, 3D printing technology is mainly applied in cutting-edge applications, such as hydrogen fuel cells and porous medical materials for drug delivery, etc.

There are several limitations in the present form of 3D printing technology, involving machines, environment, materials, and other factors. The current 3D printing product rate is only 30–50%. A relevant report points out that in 2020 with 3D printing, plastic consumption reached 18,500 tons [27]. However, there is no clarity about the amount of 3D printing waste. Probably, at least 5000 tons of 3D printing waste will be generated. 3D printing is one of the most promising manufacturing methods. In the future, it is necessary to reduce the amount of scrap and achieve a circular economy. Recycling is the most effective way to reduce printing costs. However, research on the recycling of 3D printing waste is limited. Usually, the 3D printing waste, such as PLA and ABS, is mainly treated by physical recycling methods, such as shredding and reprocessing after melting or high-temperature degradation. However, these methods are challenging for achieving efficient and green recycling.

## 4. Circular Application of 3D Printing Waste

### 4.1. Biodegradation Makes 3D Printing Materials Harmless

Nowadays, biomedicine has become the focus of research, especially 3D printed human organs. It has attracted tremendous advantages over traditional regenerative methods such as high precision cell placement, cell concentration, resolution, and precise cell diameter. Recently, a 3D bioactive scaffold has been a hot topic of research in bone tissue engineering. The technological advancement of 3D printing provides a novel and alternative approach to support bone regeneration and overcome the limitation of transitional bone grafts.

**Figure 3:**
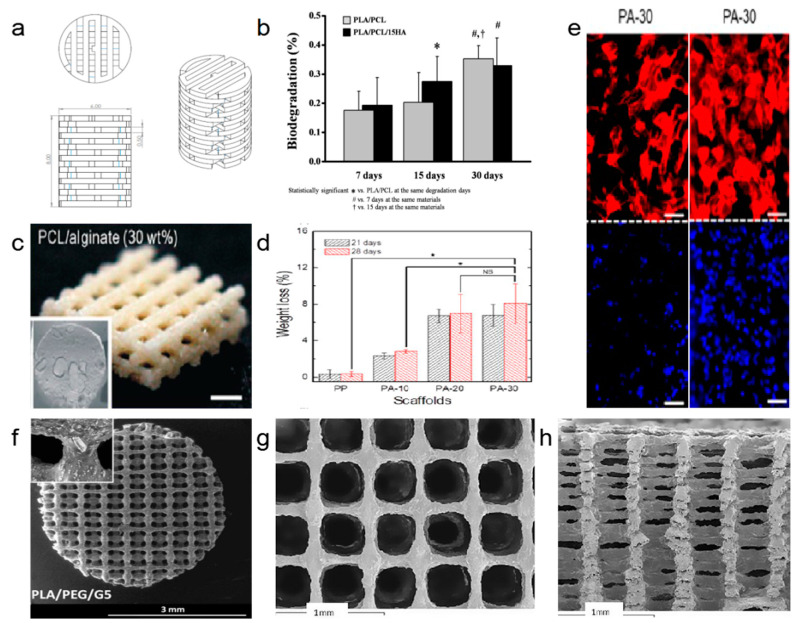
Biodegradable materials. (**a**) The shape of cartilage repair scaffold and (**b**) degradability of the cartilage repair scaffold [28]; (**c**) shape of bone scaffold [29]; (**d**) degradability of the composite with different alginate content [29]; (**e**) growth of nucleus (red) and F-actin (blue) on the scaffold at 14 (left) and 21 days (right) [29], scale bar: 50 μm; (**f**–**h**) morphology, top view, cross-sectional view of the composites with PLA, PCL, and CaP particles [31].

Generally, the biomaterials used for 3D scaffolds are natural and synthetic polymers, ceramics, and metals. Among them, biodegradable polymers like PLA are most widely utilizing biomaterials for clinical applications, especially tissue engineering and bone repair. The biodegradation rate and process of such materials depend on the composition’s crystallinity and molecular weight. A 3D printed scaffold using PCL, polyhydroxyalkanoates (PHA), and PLA composite were reported recently (Figure 3a). The scaffold generates multiple cells that are used for organ repair. Such promising outcomes of 3D printing composites believed to facilitate composite degradation in the body (Figure 3b), eliminate the removal problem, and eliminate the need for osteochondral plug harvesting [28]. In line with the above context, 3D printing bone scaffold has also been successfully applied by grounding PCL and alginate composite together, as shown in Figure 3c. The product is a three-layer structure, with layers perpendicular to each other. The prepared scaffolds showed significantly higher cell seeding efficiency, cell survival rates, good properties in water absorption, high osteoblast survival (Figure 3e), and can also degrade in vivo (Figure 3d). The weight loss is 8% at approximately 30wt% alginate content at 37 °C for 28 days. Compared to pure PCL scaffolds, cell differentiation is significantly induced [29]. In addition to bone and cartilage, many tissues and organs in the body require a scaffold to assist in regeneration. T. Serra et al. incorporated Polyethylene glycol (PEG) into PLA to form a PLA/PEG blend and prepared a 3D printed scaffold for tissue engineering application. Herein, different PEG concentrations into the PLA matrix decrease the mechanical properties of the 3D structure. However, at the same time, it improves the degradation rate of the PLA/PEG blend with increased surface morphology and wettability even at low temperatures [30]. In another study, the group reported that CaP particle addition could improve the 3D printed composites. By adding CaP, the 3D printed composite’s surface morphology changed (Figure 3f–h). CaP can enhance cytocompatibility, CaP has good biocompatibility, and it can be degraded in the human body. The scaffold prepared from PLA-CaP composite can make the cells diffuse better and help with the regeneration of muscle tissue compared to scaffolds without CAP incorporation [30,31]. Moreover, 3D printing of other parts of the body is the current research trend and is expected to be affordable soon.

In summary, from the above discussion, PLA plays a significant role as a 3D printing biomaterial in the medical field in terms of cost, performance, and other aspects. In addition to this, in the following section, various polymerization methods of PLA and their corresponding properties were studied. The solid-state polycondensation method can increase the molecular weight of PLA, reduce the residual monomer, and reduce cost. It is proved that PLA degrades highly in thermal, radiation, biological, enzymatic, and non-enzymatic degradation methods [32].

Isun3D Inc. has manufactured some 3D printed medical cryogenic thermoplastic sheets for in vitro, such as rehabilitation supports and orthopedic devices. Their products are made of PCL and can be completely degraded in soil within 8–16 months for non-pollution [33].

Any plastic has its life cycle. Although plastic can be used as fuel, its pollution when used as fuel is not negligible, and a landfill is one way to dispose of plastic, so biodegradability is important for the circular economy. Through biodegradation, the products CO_2_ and H_2_O have almost no impact on the environment. The circular economy’s ultimate goal is to reduce the environmental impact of economic activities to the lowest possible level. The reduction principle of the circular economy also includes the reduction of pollutant emissions. The harmless treatment of waste is also one way to experiment with the development of the circular economy.

### 4.2. Degradation by Catalyst/Solvent

The degradation of plastic cannot be reused more than once. Still, with the recent advancement of science and technology, the development of catalysts and solvents can promote the degradation of 3D printed products. The polymer can be degraded to the pure polymer or even monomer, but such type of degradable plastics is limited; only ester polymers can use these methods.

**Figure 4:**
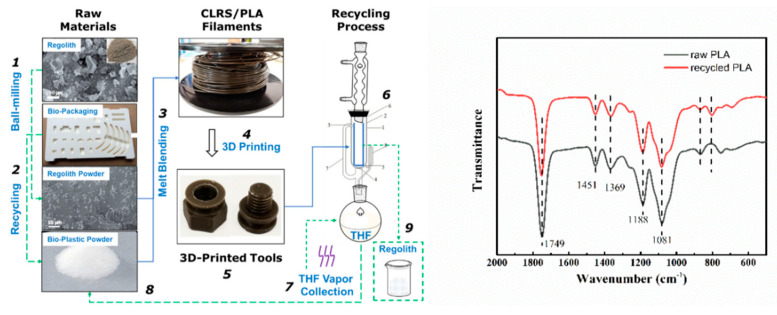
Degradation modes of polylactide/lunar regolith stimulant (PLA/CLR-1) and spectral analysis of recovered and original PLA [34].

The development of technology has led people to space, but the space resources are limited. Therefore, different 3D printing tools are needed, yet the strategy to reuse such materials is the million-dollar question. Recently, polylactide/lunar regolith stimulant (PLA/CLR-1) composite materials were reported as 3D printing materials in space by CAS. During the initial material performance, the as-prepared composite exhibits only a small reduction in mechanical properties. Moreover, to completely separate the PLA from CLR-1, the solvent dissolution method has been utilized for material recycling, as shown in Figure 4. They also observed that, in the spectroscopic analysis, the recovered PLA’s molecular weight slightly decreased, but still, it was fully reusable. Such a promising result suggests that 3D printing could be a promising tool to solve the problem with limited space resources [34].

**Figure 5:**
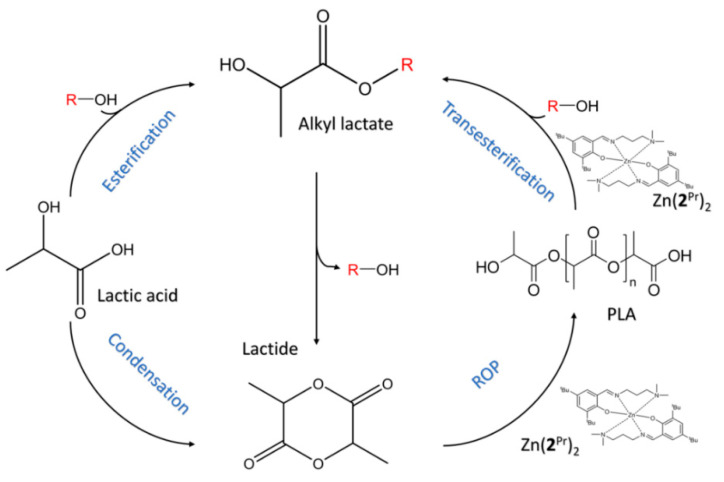
Poly(lactic acid) polymerization and depolymerization with a Zn(II) catalyst complex and their foot in a circular economy process [35].

In 3D printing materials, as mentioned above, it can be seen that PLA plays a significant role in our daily lifestyle. Although PLA is biodegradable, its degradation duration mainly relies on the environmental condition. 3D printing plastic can completely degrade in soil, but the degradation process is prolonged, and within a few weeks, the degradation will be almost null. Transforming the waste PLA 3D printing material into the small molecule, alcohol was utilized as the solvent and malondiamine Zn(II) as the catalyst. Ethyl lactate was prepared, as showed in Figure 5. Ethyl lactate is useful as a green solvent in food, pharmaceuticals. The degradation of PLA by this method depends only on the additive and the solubility of the scrap. It is possible to obtain a large yield and high selectivity quickly at a low temperature. Thus, this method has a great prospect in dealing with waste PLA 3D printing material [35].

The ionic liquid is an emerging green solvent/catalyst, non-flammable, and non-volatile, widely used in many degradation processes, especially in the degradation of polyester.

**Figure 6:**
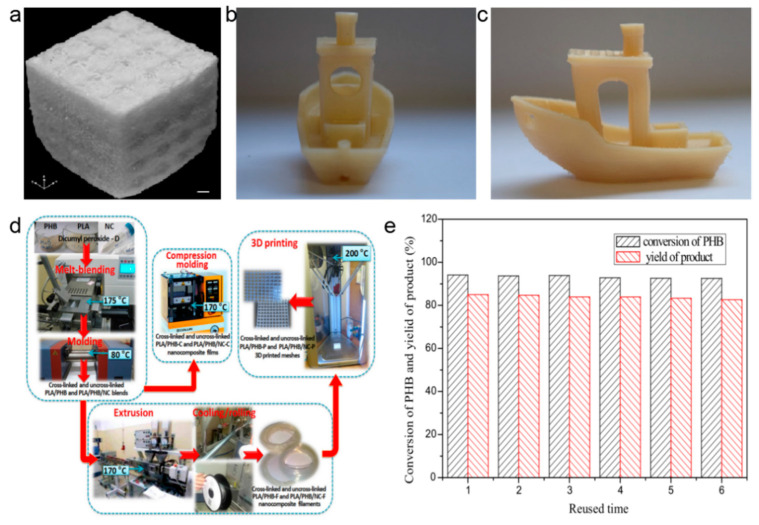
3D printing of Poly β-Hydroxybutyric (PHB) and its degradation. (**a**–**c**) PHB 3D printing patterns [38,39]; (**d**) PHB 3D printing process [40]; (**e**) reusable result of [Bmim]FeCl_4_ [42].

In the context of PLA, as discussed above, Poly β-Hydroxybutyric (PHB) is now widely studied in medicine. However, PHB as a 3D printing material was utilized less often than PLA. Figure 6a–c show some recent research using PHB as 3D printing material. PHB can be copolymerized with 3-hydroxyvalerate to form poly(3-hydroxybutyrate-co-3-hydroxyvalerate) (PHBV) [36,37] or with PLA by co-blending modification [38,39,40] to get new 3D printing materials as shown in Figure 6d. Through performance testing, these materials can be applied to in vivo scaffolds. Although PHB can be degraded by enzymes, thermal cleavage, photo-cracking, and other ways [41], there are still some disadvantages, such as slow degradation, high energy consumption of degradation, and impurity recovered material.

Therefore, ionic liquids can facilitate PHB degradation to achieve a clean product quickly. Using [Bmim]FeCl4 as the ionic liquid, the degradation of PHB was reported. Compared with [Bmim]Cl and FeCl3, [Bmim]FeCl4 has the advantage of a high degradation rate (Figure 6e), where β-Hydroxybutyric acid can be reused [42]. For comparison, they also degrade PHB with [MIMPS]FeCl4 as ionic liquid and got similar results. At present, the recycling of PHB is mainly focused on polymer degradation to obtain a pure monomer, recognized as chemical recycling. For the physical recycling of PHB, direct reuse is not commonly studied. However, the ionic liquid method can effectively reduce 3D printing waste into a useful monomer.

**Figure 7:**
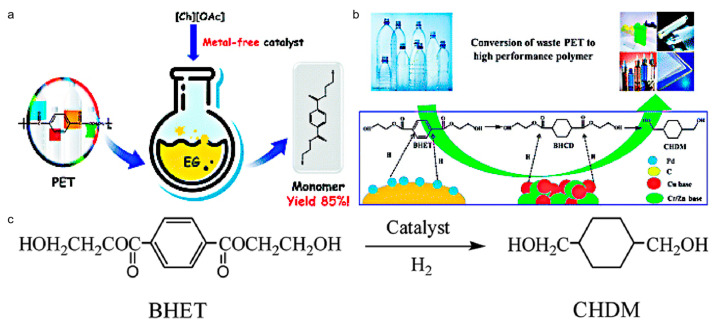
Conversion ways of PET. (**a**) Conversion of PET to bis(hydroxyalkyl) terephthalate (BHET) in ionic liquid [45]; (**b**) process from waste PET to new 1,4-cyclohexanedimethanol (CHDM) product [47]; (**c**) conversion equation from BHET to CHDM [46].

PET is an essential plastic in daily life is one of the important raw materials for 3D printing. PET’s raw material, terephthalic acid, is a non-renewable resource, so its effective recycling is crucial. In a recent study, Yu et al. reported the degradation of lipid polymers to small molecules and possessed excellent results. They derived from PET that almost all ester polymers can be degraded to small molecules by ionic liquids. Ionic liquids as catalysts can be used in PET glycolysis to convert it to useful bis(hydroxyalkyl) terephthalate (BHET) [43,44]. One of the ionic liquids is a metal-free choline-based ionic liquid, choline acetate ([Ch][OAc]) (Figure 7a). This conversion technology is cheap, better performing, and environmentally friendlier than traditional imidazolium metal-based ionic liquids [45]. [Ch][OAc] degrades the PET 3D printed plastic waste into BHET with an efficiency of up to 85.2%. In addition, it is possible to prepare 1,4-cyclohexanedimethanol (CHDM) for paints, modified materials, etc., by further reacting BHET with hydrogen (Figure 7b,c) [46,47].

According to the anions, the ionic liquid was classified into halogenated salts and novel ionic liquid (the anions are mostly BF4-, PF6-NO3-, etc.). Ionic liquids have good thermal stability and electrical conductivity. They are liquid between −100 °C and 300 °C, with low vapor pressure, and not easy to volatilize, which will not cause pollution to the atmosphere, and have good prospects in the circular economy. However, the post-treatment of ionic liquids is problematic because of its too stable and unclear toxicity nature and its impact on the environment. Therefore, more research needs to focus on studying the defects of ionic liquids when we use them as much as possible in the future.

### 4.3. Direct Reuse of 3D Printed Waste Plastics

The recycling of 3D printed plastics needs to be done by other means. In daily life, some plastics are not contaminated but are often discarded after being used once. This discarded clean plastic can be directly recycled and reused. Similarly, such waste plastic can also be used in 3D printing. Usually, repeated use of this method leads to the reduction of the plastic properties and therefore, the number of uses of this method is limited.

**Figure 8:**
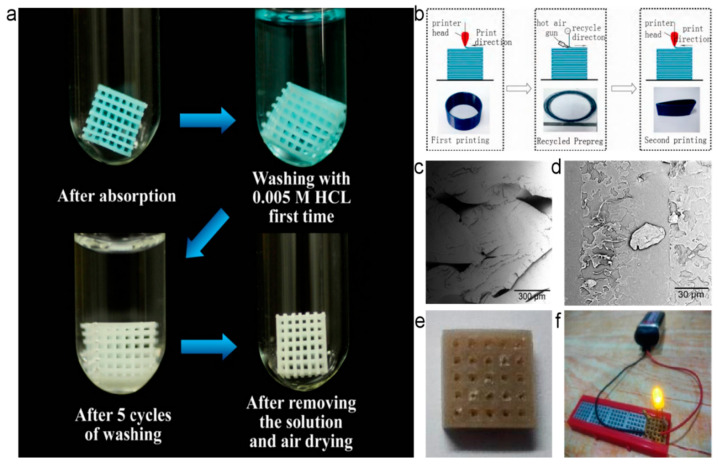
Direct reuse process diagram and products. (**a**) Recycling of Acrylonitrile Butadiene Styrene (ABS) adsorption material skeleton and adsorption process [48]; (**b**) carbon fiber recycling process of carbon fiber reinforced PLA [50]; (**c**) surface morphology of PLA composite with waste PLA [52]; (**d**) surface morphology of PLA composite with waste PLA after adding other materials [52]; (**e**,**f**) recycled PET 3D after printing as electronic devices [49].

For regular usage, the ABS is the most often used due to its toughness, cheapness, and rigidity. Recently, an ABS framework coated with Cu-BTC as a porous metal-organic framework composite has been reported for the first time by 3D printing to investigate the material adsorption. After several adsorptions of methylene blue (MB) with dilute HCL, a clean ABS skeleton material was obtained, as shown in Figure 8a. Such skeleton material can be directly reused. In terms of applications, methylene blue solution can be easily absorbed with the as-prepared kind of framework. It gives different shapes by 3D printing technology for different occasions [48]. It is also essential to study the processing and reuse of other frequently used 3D printing materials. PET is another mostly used 3D printing material. A group recently examines waste PET’s performance as a 3D printing material. The results obtained were compared with the performance of pure PET. The results proved that the recycled PET is more suitable as a 3D printing material than the pure PET. As a result, the waste 3D print PET filament was applied to prepare circuit boards [49], as shown in Figure 8e,f.

Since most plastics are difficult to degrade and negatively impact the environment, PLA has already emerged as a degradable plastic, and research focusing on 3D printing PLA plastic is spiking up. Recently, with carbon fiber and PLA, a reinforced composite material was reported. The bending strength and modulus of the composite material reach 390 MPa and 30.8 GPa. The carbon fibers could be recycled by using a heat gun to melt PLA point by point at 240 °C in the opposite direction of the print path, as shown in Figure 8b. The recycled fibers can be processed directly. Simultaneously, the PLA recycling rate is as high as 75%. The fiber recycling rate is almost 100%. The remaining 25% of PLA can be degraded, which achieved efficient recycling [50]. For the discarded PLA, the recycling process is similar to the above. It can be blended with new PLA to recover the performance loss of recycled PLA [51]. In another study, on the incorporation of epoxy-based chain extender (CE), natural fibers microcrystalline cellulose (MCC), etc. (Figure 8c,d), were used as new 3D printing materials. Although the mechanical properties are poor, the thermal properties are similar to the original material and still a good choice in recycling waste PLA 3D printing materials [52].

### 4.4. 3D printing Waste Plastic Processing and Reuse

Compared with the direct reuse in the previous section, the contaminated 3D printing waste plastics must be processed to improve their performance. As discussed in the previous section, the number of repetitions determines the performance of the modified plastic.

**Figure 9:**
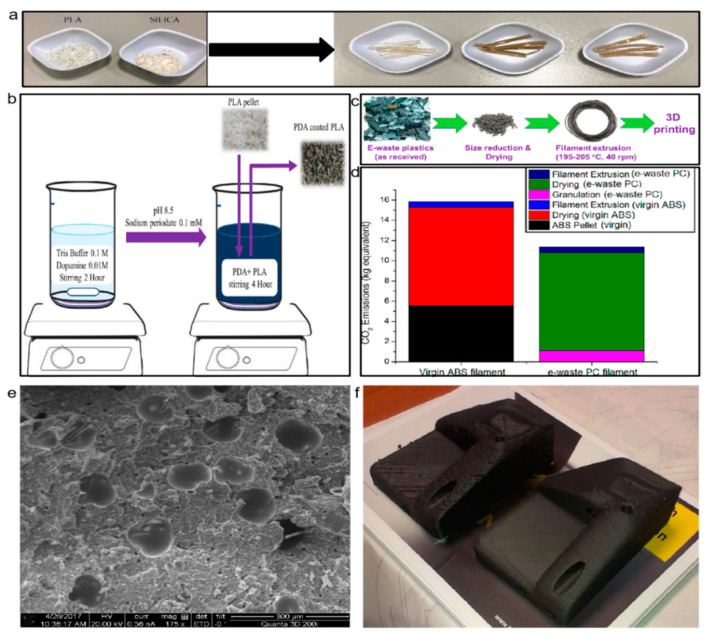
The shape of reprocessed waste plastic products. (**a**) Waste PLA blended with SiO_2_ [55]; (**b**) waste PLA blended with dopamine [56]; (**c**,**d**) recycling printing process of waste PC material and the energy consumption of waste PC compared with ABS [59]; (**e**) distribution of fillers inside PETG [57]; (**f**) on the left is the second printing of the commercial PETG filament, and the right side shows the second printing of the prepared PETG blended filament [57].

Using the fused particle fabrication (FPF) method, Woren A.L. et al. studied recycling conditions of PLA and ABS polymers and PET and PP plastics wastes. They found that the performance of recycled ABS waste and PET 3D printing waste was similar to the products printed by the fused filament fabrication (FFF) method. Moreover, the performance of PLA and PP was recorded relatively low, which can be improved by adjusting the particle size, printer aperture, and other aspects [53]. Anderson et al. reported a comparative study between new PLA 3D printed products with recycled PLA 3D printed products that were ground into a powder and then printed. They resulted that although a series of properties was slightly reduced, the overall performance is similar to the original filament. Due to the high scrap rate of 3D printing, the reuse of waste PLA decreases the costs and reduces the waste of resources [54]. Ahmed et al. studied the impact of silica additive into PLA matrix 3D printing. As shown in Figure 9a, when the silica addition amount is 10 wt%, its tensile strength, flexibility, elastic modulus, and other properties are greatly enhanced. The application of this kind of composite material is feasible, but once the silica content exceeds 15%, the performance will be reduced [55]. In addition, adding reinforcing materials, extruding 3D printed PLA waste into filaments and coating it with dopamine, as shown in Figure 9b, is also an effective way to enhance the mechanical properties of recycled PLA. Experiments have proved that the PLA waste coated by dopamine can effectively improve the tensile properties of PLA waste through chemical bonding, and it can be applied to the injection molding process [56].

For those materials that cannot be processed by simple pelletizing and extrusion, additives can add additives to make the waste plastic meet the performance requirements. Recently, Kováčová, M. Et al. developed a new type of 3D printing material-modified polyethylene phthalate (PETG) by combining PETG with graphite and carbon fiber. As shown in Figure 9e, after performance testing, it was found that the composite material has excellent performance. Still, at the same time, the recycled composite material has not significantly changed the performance after secondary printing, as shown in Figure 9f. As a result, the cost was greatly reduced, and even the quality was reduced by 25% after secondary printing. Finally, this kind of composite material has great advantages in environmental and economic aspects [57]. In the SLS print method, the most commonly used material is PA-12, which produces a lot of waste. By melting and extruding the waste with TPU or graphite, the new composite material was prepared by FDM 3D printing. After testing, it was found that there is no significant change in the performance of the used PA-12 material and the new PA-12. When compounded with TPU (10 wt%), the composite modulus increases by 1.5 times and the elongation rate increased twice the times. When compounded with graphite, the modulus of the composite material increased and can be applied to bearings and other aspects [58].

Today, engineering plastics make up for the lack of general-purpose plastics, such as engineering plastics have stronger mechanical properties, good stability, and a wide range of operating temperatures, but due to these advantages of engineering plastics, the high mechanical properties and high stability in the recycling process can make it difficult to recycle engineering plastics. Therefore, the purpose of recycling is to reuse the advantages of engineering plastics.

The widespread use of electronics in daily life has led to an increase in waste products. Recently, a study processes electronic waste products from printers through a pelletizing, drying and extrusion process (Figure 9c) to become 3D printing materials. By comparing with pure ABS printing (Figure 9d), the using waste PC as a 3D printing material reduces carbon emissions by 28%. Its breaking strength and tensile strengths are 76% and 83%, then pure ABS plastic is sufficient for practical applications. The use of waste PC plastic can be recycled for three 3D printing cycles, and the waste can be reused. They not only use waste products for 3D printing but also recycled waste from 3D printed products [59].

## 5. Recommendation

3D printing is emerging to be a powerful technology that can produce various products with unique characteristics and have more advantageous manufacturing tools than traditional manufacturing. In the future, 3D printing plays a wide range of roles in various applications, so it is necessary to achieve a circular economy.

The current difficulty in achieving a circular economy for plastics is the lack of understanding from the consumer side. Therefore, governments need to introduce the concept of the circular economy by making 3D printed products more viable.

In terms of product design, the selection of 3D printing plastics should consider the use of biodegradable plastics and minimize the use of non-degradable plastics and the production of microplastics. To boom the 3D printing industry, the government should need to provide financial support and the promotion of the circular economy to achieve the use and development of materials more conducive to recycling. In addition, individual companies should pay attention to the importance of the recycling industry, which generates far more profit than industries that use primary resources for production. In terms of business model, the Italian company Montello S.P.A., which has moved from the traditional steel industry to a green economy industry, was the first Italian company to use fully automated technology to recycle plastic packaging. Every year, 80% of the recycled waste plastic is converted into secondary raw materials and 20% into solid fuel. Finally, 3D printing companies should learn from Montello S.P.A. to recycle and reuse their products to achieve a circular economy.

## 6. Conclusions

Today, the most conventional ways to dispose of waste 3D printing plastics are in landfills as well as burning, which is not wise because most plastics degrade very slowly, and burning poses a greater environmental risk. In this case, the reuse or degradation of 3D printing plastics is the best choice to deal with 3D printing waste plastics.

At present, 3D printing plastic recycling methods include biodegradation, adding catalyst/solvent for recycling, direct reuse, and processing and reuse. For degradation, although it can be pollution-free, it takes a long time. For the catalyst/solvent method, although recycling can be degraded to monomer reuse, usually only lipid polymers can use this method, and the suitability of the solvent requires experimental verification. The direct reuse method requires high-quality waste plastics, and the types of plastics that can be directly recycled are limited. The method of processing and reuse requires additional energy to reprocess. Both direct reuse and processing and reuse methods may cause the problem of product performance degradation.

The choice of plastic-type plays an important role in the circular economy of plastics. If degradable ester plastics are selected, ionic liquids can be chosen as catalysts for the treatment, offering better selectivity of end products, and degradation efficiency of over 80 wt% for ester polymers. In addition, the ionic liquid is a “recycling” catalyst, which can be used multiple times, in line with the concept of the circular economy. When these plastics are too polluted to be catalytically degraded, they can be landfilled directly, and the CO_2_ and H_2_O produced are virtually harmless to the environment. For some of the cases, where ester plastics are not demandable, recycling is an option when other plastics are used. Still, the number of cycles of these plastics is equivalent to their lifetime, shorter than the catalyst degradation. The reuse of these plastics is also limited by the level of contamination of the plastic.

In conclusion, future research on 3D printed plastics for the circular economy should regard more types of 3D printed plastics and discuss how these plastics should be processed concerning their properties, contamination level, and the number of repetitions.

## Figures and Tables

**Table 1:** The difference between 3D printing from traditional molding processes.

Advantages	Describe
Manufacturing complex items without increasing costs	In traditional manufacturing, the more complex the shape of the object, the more expensive it is to manufacture. For 3D printers, the cost of manufacturing complex-shaped objects does not increase. Making a gorgeous complex-shaped object does not consume more time, skill, or cost than printing a simple cube.
Product diversity	A 3D printer can print many shapes. Traditional manufacturing equipment has fewer features and makes a limited variety of shapes.
Human resource	A 3D printer requires only different digital design blueprints and a new batch of raw materials and do not need specialized staff.
No assembly required	3D printing enables parts to be molded in an integrated manner. Traditional mass production is based on assembly lines, and in modern factories, machines produce identical parts and then are assembled by robots or workers (even across continents).
Deliver immediately	3D printers can print on demand. Just-in-time production reduces a company’s inventory, and companies can use 3D printers to create special or customized products to meet customer needs based on customer orders.
Small footprint, easy to carry	In terms of manufacturing space per unit, 3D printers have greater manufacturing capabilities than traditional manufacturing machines. The machine can move freely, which makes them suitable for home or office use.
Infinite combinations of materials	Traditional manufacturing machines cannot easily fuse multiple raw materials during the cutting or mold forming process, but 3D printing can do it.

**Table 2:** Types of 3D printing methods and corresponding material requirements.

Methods	Processing	Material Requirements
SLA	Cure the polymer by light.	Viscosity, flexibility, moisture resistance, and fracture energy of the material after molding.
LOM	Laminate the material and cut the sheet.	Need to have high heat resistance, high hardness, mechanical properties, etc.
FDM	Melt the material in the apparatus and spray the melt.	Viscosity and melting point.
SLS	Sinter the powder, layer by layer, to form the products.	Need powder material.
3DP	Bond the sprayed powder.	Gypsum.

**Table 3:** Types of 3D printing plastics required as well as their advantages and disadvantages.

Material	Material Requirements	Advantages	Disadvantages
1. ABS 2. PLA 3. PCL 4. PA 5. PC, etc.	Good processing performance; material performance should be suitable for the occasions.	A wide range of products	More expensive, some other properties are sacrificed to ensure processing performance [24]

## Data Availability

The data presented in this study are available on request from the corresponding author.
